# In Vitro Analysis of the Fatigue Resistance of Four Single File Canal Preparation Instruments

**DOI:** 10.3390/ma15020688

**Published:** 2022-01-17

**Authors:** Mohammad I. Al-Obaida, Abdulmohsen A. Alzuwayer, Saqer S. Alanazi, Abdulrahman A. Balhaddad

**Affiliations:** 1 Department of Restorative Dental Sciences, College of Dentistry, King Saud University, P.O. Box 60169, Riyadh 11545, Saudi Arabia; malobaida@ksu.edu.sa; 2 Dr. Abdulaziz Al Ajaji Dental Polyclinic, Riyadh 12836, Saudi Arabia; Dr.alzuwayer@gmail.com; 3 Department of Dentistry, Al-Kharj Military Industries Corporation Hospital, P.O. Box 318, Al Kharj 11942, Saudi Arabia; saqeralenazy@gmail.com; 4 Department of Restorative Dental Sciences, College of Dentistry, Imam Abdulrahman Bin Faisal University, Dammam 31441, Saudi Arabia

**Keywords:** endodontic, fracture, reciprocation, root canal therapy

## Abstract

Instrument separation during root canal therapy is inevitable in endodontics with several unfavorable clinical consequences. Therefore, examining the cyclic flexural fatigue resistance of commonly used rotary endodontic files is crucial. This study aimed to determine the cyclic flexural fatigue resistance of four nickel–titanium (NiTi) rotary files used as a single canal preparation technique: WaveOne, Reciproc, Protaper F2, and Unicone medium instruments. According to the manufacturer’s instructions, each file was rotated freely within a 1.3 mm deep and 1.3 mm wide V-shaped groove in a stainless-steel block with a 40° and 5 mm radius of curvature. Cyclic fatigue resistance was compared between the NiTi files by verifying the time needed to crack. The data were analyzed using one-way analysis of variance (ANOVA) followed by Scheffé post hoc with a significant level set at *p* < 0.05. Our results demonstrated that the WaveOne instrument had the highest cyclic flexural fatigue resistance among the tested groups (*p* ≤ 0.05), while Unicone had the lowest cyclic flexural fatigue resistance. This study concluded that WaveOne size 25/0.08 could illustrate a superior cyclic flexural fatigue resistance when instrumenting root canals with the lowest possibility to cause instrument separation.

## 1. Introduction

In endodontics, the amputation of pulpal tissues and the removal of microorganisms responsible for the infection are the essential objectives of root canal therapy [1]. Root canal therapy attempts to achieve optimal disinfection by removing the infected tooth structure within the root canal system using mechanical and chemical approaches [2]. The mechanical tools include using endodontic files to clean and shape the canals. Stainless-steel (SS) hand files have been used for many decades in cleaning and shaping root canal systems [3]. However, several drawbacks have been reported with the use of SS files, such as unintentional changes in the canal walls during the cleaning and shaping process, which may complicate obturating the root canal system appropriately [4].

To overcome the obstacles associated with SS files, nickel–titanium (NiTi) materials have been introduced in dentistry. NiTi endodontic files are less likely to induce undesirable alterations or procedural inaccuracies while preparing the canals [5,6]. NiTi was first invented in the 1960s when Buchler et al. created a superelastic alloy composed of nickel and titanium, termed “Nitinol” [7]. Nitinol has the capability to recover its actual shape following elastic deformation [8], making it a perfect material to clean and shape severely curved root canals [9]. Using NiTi alloys to synthesize endodontic files was first proposed by Civjan et al. [10]. Then, Walia et al. had the idea to attempt a NiTi endodontic file, demonstrating this material’s high elasticity and resistance when subjected to twisting and torsion, compared with SS files [11]. 

Despite the numerous advantages, separation of the NiTi instrument may occur without any signs of torsion or flexion [12]. Instrument separation occurs when the NiTi instrument surpasses the standard elastic limit [13]. During the rotational movement inside curved canals, the instrument is kept in a stationary condition at the apical constriction of the canal, while the coronal part maintains its rotational movement [14,15]. As a result, a part of the instrument’s shaft on the outside of the curve is in tension when the other part of the shaft is inside the curve. This may cause compressive tension stress resulting in cyclic fatigue [16]. The repeated tension compressions in curved canals increase the incidences of instrument separation [14,15].

Instrument separation is a significant issue in endodontics as the separated instrument may interfere with cleaning and shaping the canal, compromising the disinfection procedure [17]. Moreover, the separated instrument can result in under-filling of the treated canal, which may affect the apical seal of the root filling [18]. All of these factors may compromise the clinical longevity of root canal therapy [18], as reduced periapical healing in root canals was observed with the incidences of separated instruments [19]. Furthermore, while removing the separated instrument is a clinical option, it could be complicated when the separation happens apically with a considerable risk of canal perforation or root fracture [20,21].

Considering this clinical issue, it is essential to investigate the cyclic fatigue resistance of commonly used NiTi rotary endodontic files. Previous investigations examined the flexural fatigue resistance of several rotary systems. However, only a few articles investigated the flexural fatigue of files used for the single canal preparation technique [22,23]. These articles compared two or three single canal rotary files. To our knowledge, no study has investigated the flexural fatigue of Unicone rotary files in the single canal technique. To obtain a more comprehensive evaluation of the most commonly used single canal instruments, this study compares the fatigue resistance and fracture time of four different single file canal preparation instruments: WaveOne M-wire NiTi, Protaper F2 NiTi, Reciproc NiTi, and Unicone NiTi files. The null hypothesis is that the recruited rotary system demonstrates similar flexural fatigue behaviors.

## 2. Materials and Methods

### 2.1. Study Design and Groups 

This study consists of four NiTi rotary endodontic files: (1) WaveOne M-wire NiTi file (Dentsply Sirona, Baillagues, Switzerland), (2) Protaper F2 NiTi file (Dentsply Maillefer, Ballaigues, Switzerland), (3) Reciproc NiTi file (RPC; VDW, Munich, Germany), and (4) Unicone NiTi file (MEDIN, Nove Mesto na Morave, Czech Republic). Table 1 shows more details concerning the NiTi rotary files. All files were 25 mm in length, with a tip size of 25 (*n* = 20). 

### 2.2. Monitoring the Cyclic Flexural Fatigue Resistance 

**Figure 1:**
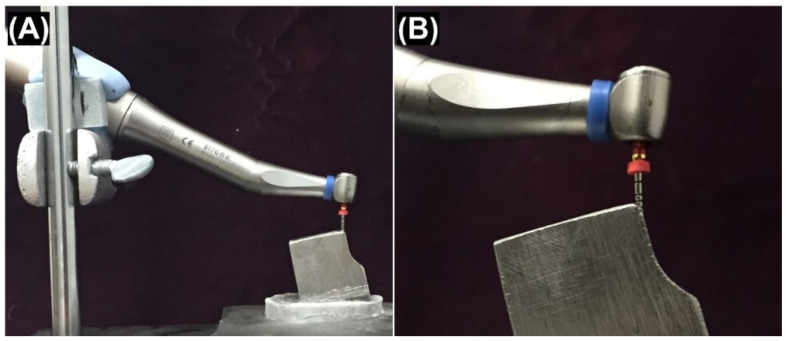
An illustration of the experimental setting involving a NiTi rotary file and the testing block. (**A**) Each NiTi file was mounted in the same pattern to standardize the procedure and to maintain contact with the testing block. (**B**) The testing block contacted a mounted NiTi rotary file while the handpiece was operating to estimate each file’s fracture time.

The files were subjected to free rotation motion within a SS block (Micron, Riyadh, Saudi Arabia) containing a 1.3 mm deep and 1.3 mm wide V-shaped groove with a 40-degree and 5 mm radius of curvature (Supplementary Materials S1), as was described previously by Schneider [24]. This device was initially designed by Haïkel et al. [25]. The tip of each NiTi rotary file was introduced 16 mm into the groove. Each testing run consists of four instruments, one file from each group (Figure 1). The type of motion for each file is shown in Table 1, including reciprocal movement for WaveOne, Reciproc, and Unicone and continuous rotation for Protaper F2. This device was constructed to develop an accurate relationship between the cyclic flexural fatigue-testing block and the contra-angle handpiece, which was connected to a machine holder at a specific position to maintain a standardized starting position throughout the study.

An air conductor attached to the device’s base directed to the groove in the testing block was used as a coolant. At a room temperature of 25 °C, each file was operated following the manufacturer’s instructions using a 6:1 reduction Sirona contra-angle handpiece (Sirona Dental Systems GmbH, Bensheim, Germany) powered by a torque-controlled electric motor (Dentsply Sirona, Baillagues, Switzerland) until an instrument fracture was visually observed. A stopwatch was used to estimate the time required for the separation of files. One operator (A.A.A.) handled the experiment set to standardize the process and condition among the groups.

### 2.3. Statistical Analysis

SPSS Pc+ version 21.0 (2018, IBM, Armonk, NY, USA) statistical software was utilized to determine the appropriate sample size and to analyze the data. One-way analysis of variance (ANOVA) was used to compare the mean values of fracture time across the four files. Scheffé post hoc test was used for pairwise comparison of mean values. A *p*-value of <0.05 was used to report the statistical significance of the values.

## 3. Results

**Figure 2:**
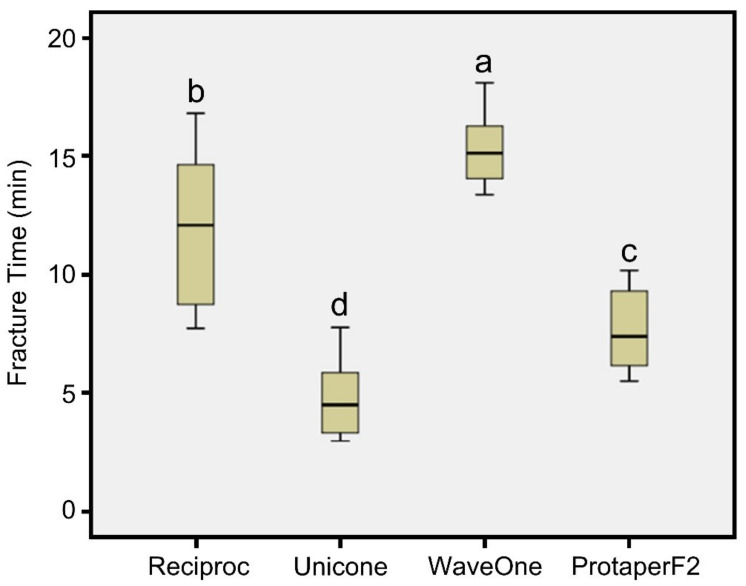
Box plot comparing the fracture time of the four endodontic rotary files. The box median is shown as a line, as are the upper and lower quartiles. Values indicated by different letters are statistically different from each other (*p* < 0.05).

Table 2 illustrates that the comparison of the mean fracture time between the tested groups was significant (F = 110.02, *p* < 0.0001; power of analysis = 100%). By using the Scheffé post hoc test, it was observed that the mean fracture time of Unicone files was significantly lower than the mean fracture time of the other three files (Protaper F2, Reciproc, and Wave One). Opposingly, the mean fracture time of the WaveOne file was significantly the highest among the groups (Figure 2). Based on this analysis, it can be concluded that the WaveOne file provided the highest resistance, while the Unicone file had the lowest resistance. The other two files, Protaper F2 and Reciproc, displayed moderate resistance to cyclic flexural fatigue. The separation occurred at the curvature site for all of the files, as this site expressed the highest amount of fatigue. This site was 3- to 4-mm away from the file’s tip, and the fracture was visualized as clean-cut and horizontal.

## 4. Discussion

Biomechanical debridement of the entire root canal system is the ultimate objective of root canal therapy. Biomechanical debridement aims to eradicate the damaged pulp tissues and the pathogenic microorganisms within the root canal structure and to provide an ideal environment for final obturation [26]. The NiTi rotary instrument has superelastic behavior, making it one of the advanced tools in root canal preparation [11]. The reciprocation movement can play a prominent role in reinforcing cyclic fatigue life [22]. The risk of more significant instrument separation is due to decreased flexibility, which overlaps with the instrument’s performance in cyclic fatigue separation resistance [22]. Our study found that the WaveOne rotary file system had better cyclic flexural fatigue resistance compared with other systems, which was associated with fewer separation incidences during root canal therapy.

Roane et al. introduced the “balanced force” technique in 1985 [27], which involved three main steps. First, after introducing the instrument passively in the canal, a 90-degree clockwise rotation was performed to involve radicular dentin. Then, the second step was achieved by rotating the file in a counterclockwise motion and with the appropriate amount of axial force. This rotational movement allowed for the breaking of the dentinal walls. In the third step, the file was retracted from the canal via a clockwise rotation [27]. However, this balanced force technique was associated with a high risk of straightening the treated canal, as the files used in this technique were not pre-curved [28].

In 2008, G Yard, who introduced the single file canal preparation technique, used F2 Protaper NiTi files instead of several hand and rotary files [25]. The F2 Protaper was attached to a 16:1 reduction ratio contra-angle handpiece connected to ATR vision, allowing for reciprocating movement [29]. This novel canal preparation system offered two significant advantages. First, it was more cost-effective than the traditional multiple files system. Second, it had a lower susceptibility to cross-contamination [29]. In addition, the files needed good elasticity and high cyclic fatigue resistance to perform optimally inside curved canals with a minimum risk of instrument fracture or separation [30]. The new concept of using one instrument in reciprocation movement for cleaning and enlarging the canal regardless of its width and curvature was working against the conventional method that needed multiple instruments to gradually enlarge the canal and to gain the final shape and size. This new technique was cost-effective and easy to master, which encouraged many dental practitioners to consider using it [29,31].

Cyclic fatigue is caused by work hardening and metal fatigue during the flexure of an instrument that is rotating freely in root canals until separation, especially in severely curved canals [32]. The fracture happens when the endodontic file exceeds its maximum flexure point. It is commonly thought that cyclic fatigue is a major factor in the fracture of endodontic rotary files utilized in a clinical setting [33]. It has been suggested that the reciprocal movement of NiTi files is associated with improved resistance against cyclic fatigue compared with continuous movement, resulting in enhanced instrument life [34]. The file achieves an anticlockwise rotation at a wide range of reciprocating movements, followed by a limited clockwise rotation [35]. In our investigation, NiTi files operating in the reciprocal movement, WaveOne and Reciproc R25, were associated with better resistance against cyclic fatigue failure than Protaper F2 operating in the continuous movement. However, Unicone operating in the reciprocal movement was inferior to Protaper F2, suggesting the involvement of other factors contributing to fatigue failure.

This study compared the resistance to cyclic fatigue of four NiTi rotary files used as a single file canal preparation. These four NiTi files were selected because they have a comparable taper, size, and the same clinical indication. This study proved that WaveOne has superior resistance to cyclic flexural fatigue, while Unicone demonstrated the lowest resistance to cyclic flexural fatigue. Our study findings are in disagreement with the results of Kim et al. [23], who assessed cyclic and torsional fatigue of WaveOne primary and Reciproc R25 with Protaper F2 by using a model composed of tempered steel with a 0.6 mm apical diameter, a 6.06 mm radius, and a 45-degree angle of curvature. Their study revealed that Reciproc had the best cyclic fatigue resistance, while Protaper F2 had the lowest value. Conversely, WaveOne had superior torsional fatigue followed by Reciproc and Protaper F2. In addition, De-Dues et al. had dissimilar results to this study [22]. Using an SS tube model, they evaluated bending resistance as well as cyclic and static fatigue of WaveOne and Reciproc R40 instruments. It was found that WaveOne had substantially superior bending resistance compared with Reciproc. However, in both dynamic and static tests, Reciproc showed extended cyclic fatigue life than WaveOne [22].

Studies used in evaluating cyclic flexural fatigue have a different set up for testing. Some of these analyses utilized steel groove [25], while others employed steel slopes [36] or three-point steel pins [37]. Moreover, the radius and angle of curvature directly affect the result of evaluating cyclic flexural fatigue of rotary instruments. In this experiment, our testing device was applied following the design of Haïkel et al. [25], but with minor alterations. The observable differences in the results are explained by the lack of international standards regarding the devices used in similar types of in vitro experiments [38].

For this study’s objective, it was ideal to use natural teeth as a model and to evaluate the cyclic fatigue resistance of the NiTi files, which was performed in some studies [39,40]. However, this was impossible to conduct due to the variations between the root canals from one tooth to another. Therefore, a designed SS block model standardized the analysis and eliminated other failure mechanisms rather than cyclic fatigue. Future investigations may consider other testing conditions and variables, such as different files’ diameters, as larger diameter files are more subjected to break due to cyclic fatigue than their smaller counterparts [14,25].

The limitation of this experiment was using Unicone in a moderate angle of curvature as mentioned in the Materials and Methods section, which is 40 degrees, which is not recommended by the manufacturer’s instructions since it lessens the cyclic flexural fatigue resistance of the file. Therefore, the suggestion for further research is to modify the angle of curvature in the testing device according to the manufacturer’s recommendation. This study did not evaluate the flumes’ wear of each file using scanning electron microscopy images, which could be considered in future analysis. Even though this study investigated the flexural fatigue of the most used single canal rotary files, future investigations may consider using other commercially available NiTi rotary files.

## 5. Conclusions

The findings of this study demonstrated that the WaveOne medium instrument had increased endurance against cyclic flexural fatigue in single canal preparation over Unicone, Reciproc, and F2 Protaper files. As a result, the WaveOne endodontic rotary file may decrease the chance of file separation when instrumenting curved canals, emphasizing the importance of its use in constricted and curved root canal systems.

## Figures and Tables

**Table 1:** Manufacturers of the four endodontic files.

Endodontic Rotary File	Material	Type of Motion	D0 Size (mm)	D0 Taper (%)	Speed (RPM)	Torque (N·cm)
WaveOne (Dentsply Sirona, Baillagues, Switzerland)	NiTi	Reciprocal movement	0.25	8	350	2
Reciproc (RPC; VDW, Munich, Germany)	NiTi M-Wire alloy	Reciprocal movement	0.25	8	300	2
Unicone (MEDIN, Nove Mesto na Morave, Czech Republic)	Niti	Reciprocal movement	0.25	8	300	3.1
Protaper F2 (Dentsply Maillefer, Ballaigues, Switzerland)	NiTi	Continuous rotation	0.25	8	150–300	1.5–3

**Table 2:** The cyclic flexural fatigue resistance by recording the time to failure in minutes (means ± SD) of each file while the speed is constant (*n* = 20). Values indicated by different letters are statistically different from each other (*p* < 0.05).

Time to Failure in Minutes	*p*-Value	F-Value	N	Groups
(Mean ± SD)				
15.37 ± 1.48 ^a^			20	WaveOne (25/08)
11.88 ± 2.98 ^b^	<0.05	110.02	20	Reciproc (R25)
4.77 ± 1.41 ^c^			20	Unicone (25/06)
7.69 ± 1.62 ^d^			20	Protaper (F2)

## Data Availability

The data set generated and analyzed in this study is available upon reasonable request to the corresponding author.

## Supplementary Materials

The following supporting information can be downloaded at: https://www.mdpi.com/article/10.3390/ma15020688/s1, A video showing a file mounted contacting the testing SS block to estimate the file’s fracture time.
